# (4-Ethenylphen­yl)diphenyl­phosphine selenide

**DOI:** 10.1107/S1600536812034289

**Published:** 2012-08-11

**Authors:** Zanele H. Phasha, Sizwe Makhoba, Alfred Muller

**Affiliations:** a Research Center for Synthesis and Catalysis, Department of Chemistry, University of Johannesburg (APK Campus), PO Box 524, Auckland Park, Johannesburg, 2006, South Africa

## Abstract

In the title mol­ecule, C_10_H_17_PSe, the P atom has a distorted tetra­hedral environment resulting in an effective cone angle of 165°. The benzene ring makes dihedral angles of 70.04 (8) and 77.28 (8)° with the phenyl rings, while the dihedral angle between the phenyl rings is 62.95 (8)°. In the crystal, mol­ecules are linked by C—H⋯π inter­actions.

## Related literature

For background to our investigation of the steric and electronic effects of group 15 ligands, see: Roodt *et al.* (2003 ▶); Muller *et al.* (2006 ▶, 2008 ▶). For background to cone angles, see: Bunten *et al.* (2002 ▶); Tolman (1977 ▶); Otto (2001 ▶).
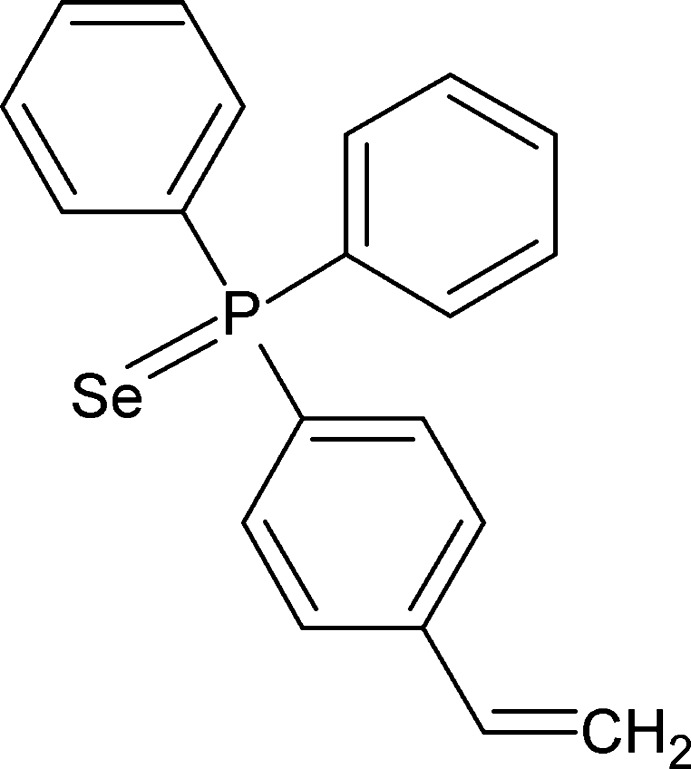


## Experimental

### 

#### Crystal data

C_20_H_17_PSe
*M* *_r_* = 367.27
Monoclinic, 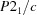

*a* = 10.5310 (7) Å
*b* = 11.1477 (7) Å
*c* = 17.2187 (9) Å
β = 124.562 (3)°
*V* = 1664.66 (18) Å^3^
*Z* = 4
Mo *K*α radiation
μ = 2.35 mm^−1^
*T* = 100 K
0.3 × 0.25 × 0.13 mm

#### Data collection

Bruker APEX DUO 4K-CCD diffractometer
Absorption correction: multi-scan (*SADABS*; Bruker, 2008 ▶) *T*_min_ = 0.526, *T*_max_ = 0.737
20089 measured reflections
4166 independent reflections
3921 reflections with *I* > 2σ(*I*)
*R*_int_ = 0.028

#### Refinement

*R*[*F* ^2^ > 2σ(*F* ^2^)] = 0.024
*wR*(*F* ^2^) = 0.063
*S* = 1.03
4166 reflections
199 parameters
H-atom parameters constrained
Δρ_max_ = 1.37 e Å^−3^
Δρ_min_ = −0.41 e Å^−3^

### 

Data collection: *APEX2* (Bruker, 2011 ▶); cell refinement: *SAINT* (Bruker, 2008 ▶); data reduction: *SAINT* and *XPREP* (Bruker, 2008 ▶); program(s) used to solve structure: *SHELXS97* (Sheldrick, 2008 ▶); program(s) used to refine structure: *SHELXL97* (Sheldrick, 2008 ▶); molecular graphics: *DIAMOND* (Brandenburg & Putz, 2005 ▶); software used to prepare material for publication: *WinGX* (Farrugia, 1999 ▶).

## Supplementary Material

Crystal structure: contains datablock(s) global, I. DOI: 10.1107/S1600536812034289/is5175sup1.cif

Structure factors: contains datablock(s) I. DOI: 10.1107/S1600536812034289/is5175Isup2.hkl

Supplementary material file. DOI: 10.1107/S1600536812034289/is5175Isup3.cml

Additional supplementary materials:  crystallographic information; 3D view; checkCIF report

## Figures and Tables

**Table 1:** Hydrogen-bond geometry (Å, °) *Cg*1 and *Cg*2 are the centroids of the C1–C6 and C15–C20 rings, respectively.

*D*—H⋯*A*	*D*—H	H⋯*A*	*D*⋯*A*	*D*—H⋯*A*
C18—H18⋯*Cg*1^i^	0.95	2.62	3.383 (2)	137
C3—H3⋯*Cg*2^ii^	0.95	2.88	3.5889 (19)	133
C12—H12⋯*Cg*2^iii^	0.95	2.85	3.614 (2)	138

Symmetry codes: (i) 
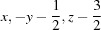
; (ii) 
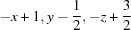
; (iii) 
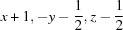
.

**Fig. 1.**
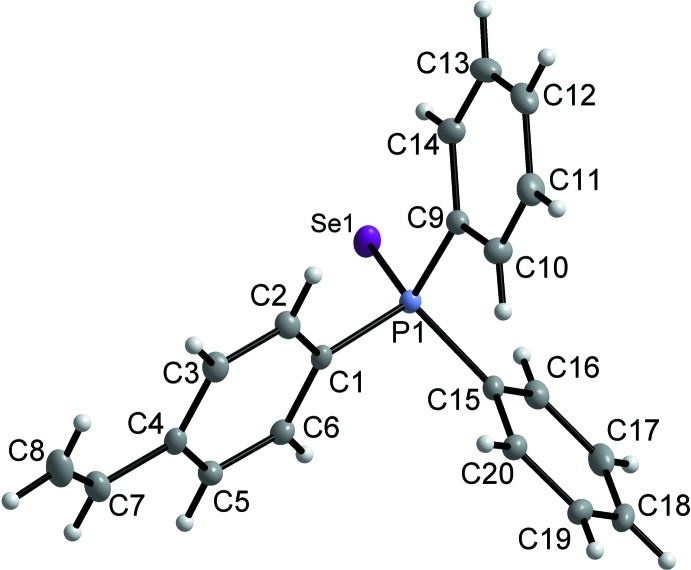
The molecular structure of the title compound. Displacement ellipsoids are drawn at the 50% probability level.

**Fig. 2.**
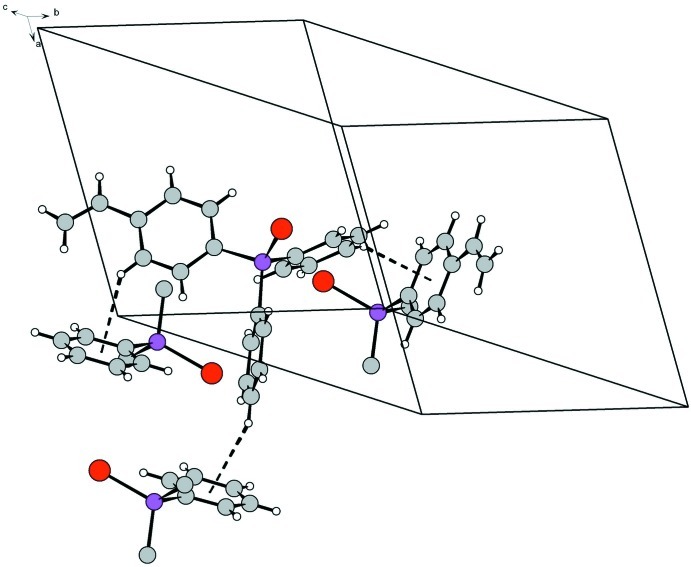
A packing diagram of the title compound, showing the C—H···π interactions.

## supplementary crystallographic information

### Comment

Various techniques such as crystallography, multi nuclear NMR and IR have been used to extensively study the transition metal phosphorous bond (Roodt *et al.*, 2003). As part of this systematic investigation we have extended this study to selenium derivatives of the phosphorus ligands (see Muller *et al.*, 2008). This way there is no steric crowding effect, albeit crystal packing effects, as normally found in transition metal complexes with bulky ligands, *e.g.* in *trans*-[Rh(CO)Cl{P(OC_6_H_5_)_3_}_2_] cone angles variation from 156° to 167° was observed for the two phosphite ligands (Muller *et al.*, 2006). The ^1^*J*(^31^P-^77^Se) coupling can also be used as an additional probe to obtain more information regarding the nature of the phosphorous bond. Reported as part of the above continuing study, the single-crystal structure of the phosphorus containing compound, SePPh_2_(4-C_2_H_3_—C_6_H_4_), where Ph = C_6_H_5_ and 4-C_2_H_3_—C_6_H_4_ = 4-vinylbenzene, is reported here.

The title compound (Fig. 1) adopts a distorted tetrahedral arrangement about the P atom with average C—P—C and Se—P—C angles of 105.74 and 112.98°, respectively. Describing the steric demand of phosphine ligands has been the topic of many studies and a variety of models have been developed (Bunten *et al.*, 2002). Of these the Tolman cone angle (Tolman, 1977) is still the most commonly used model. Applying this model to the geometry obtained for the title compound (and adjusting the Se—P bond distance to 2.28 Å as described by Tolman), we calculated an effective cone angle from the geometry found in the crystal structure of 165° (Otto, 2001). Intermolecular C—H···π interactions (Table 1 and Fig. 2) are observed in the crystal.

### Experimental

Diphenylphosphino styrene and KSeCN were purchased from Sigma-Aldrich and used without purification. Eqimolar amounts of KSeCN (5.8 mg, 0.04 mmol) and the diphenylphosphino styrene (11.5 mg, 0.04 mmol) were dissolved in the minimum amounts of methanol (10 ml). The KSeCN solution was added drop wise (5 min.) to the phosphine solution with stirring at room temperature. The final solution was left to evaporate slowly until dry to give crystals suitable for a single-crystal X-ray study.

Analytical data: ^1^H NMR (CDCl_3_, 400 MHz): δ 7.74–7.68 (m, 6H), 7.45–7.42 (m, 8H), 6.75–6.68 (m, 1H), 5.82(d,*J* = 17.6 Hz, 1H), 5.36 (d, *J* = 4.8 Hz, 3H); ^13^C {H} NMR (CDCl_3_, 400 MHz) δ 135.8,116.7 (ethylene), 133.0,132.9,132.7,132.6,131.6,128.6,128.5,126.3,126,2 (Ar) ^31^P {H} NMR (CDCl_3_, 161.99 MHz): δ = 34.71 (t, ^1^*J*(^31^P-^77^Se) = 729 Hz)

### Refinement

All hydrogen atoms were positioned in geometrically idealized positions with C—H = 0.95 Å (aromatic and methylene), and allowed to ride on their parent atoms with *U*_iso_(H) = 1.2*U*_eq_(C). The highest residual electron density of 1.37 e.Å^-3^ and the deepest hole of 0.41 e.Å^-3^ are both located within 1 Å from Se1. Both represent no physical meaning.

### Crystal data

**Table:**

C_20_H_17_PSe	*F*(000) = 744
*M**_r_* = 367.27	*D*_x_ = 1.465 Mg m^−^^3^
Monoclinic, *P*2_1_/*c*	Mo *K*α radiation, λ = 0.71073 Å
Hall symbol: -P 2ybc	Cell parameters from 9906 reflections
*a* = 10.5310 (7) Å	θ = 2.5–28.4°
*b* = 11.1477 (7) Å	µ = 2.35 mm^−^^1^
*c* = 17.2187 (9) Å	*T* = 100 K
β = 124.562 (3)°	Plate, colourless
*V* = 1664.66 (18) Å^3^	0.3 × 0.25 × 0.13 mm
*Z* = 4	

### Data collection

**Table:**

Bruker APEX DUO 4K-CCD diffractometer	4166 independent reflections
Graphite monochromator	3921 reflections with *I* > 2σ(*I*)
Detector resolution: 8.4 pixels mm^-1^	*R*_int_ = 0.028
φ and ω scans	θ_max_ = 28.4°, θ_min_ = 2.3°
Absorption correction: multi-scan (*SADABS*; Bruker, 2008)	*h* = −14→14
*T*_min_ = 0.526, *T*_max_ = 0.737	*k* = −14→14
20089 measured reflections	*l* = −16→22

### Refinement

**Table:**

Refinement on *F*^2^	Primary atom site location: structure-invariant direct methods
Least-squares matrix: full	Secondary atom site location: difference Fourier map
*R*[*F*^2^ > 2σ(*F*^2^)] = 0.024	Hydrogen site location: inferred from neighbouring sites
*wR*(*F*^2^) = 0.063	H-atom parameters constrained
*S* = 1.03	*w* = 1/[σ^2^(*F*_o_^2^) + (0.032*P*)^2^ + 1.0912*P*] where *P* = (*F*_o_^2^ + 2*F*_c_^2^)/3
4166 reflections	(Δ/σ)_max_ = 0.002
199 parameters	Δρ_max_ = 1.37 e Å^−^^3^
0 restraints	Δρ_min_ = −0.41 e Å^−^^3^

### Special details

**Table:**

Experimental. The intensity data was collected on a Bruker Apex DUO 4 K CCD diffractometer using an exposure time of 1.5 s/frame. A total of 1478 frames were collected with a frame width of 0.5° covering up to θ = 28.40° with 99.9% completeness accomplished.
Geometry. All e.s.d.'s (except the e.s.d. in the dihedral angle between two l.s. planes) are estimated using the full covariance matrix. The cell e.s.d.'s are taken into account individually in the estimation of e.s.d.'s in distances, angles and torsion angles; correlations between e.s.d.'s in cell parameters are only used when they are defined by crystal symmetry. An approximate (isotropic) treatment of cell e.s.d.'s is used for estimating e.s.d.'s involving l.s. planes.
Refinement. Refinement of *F*^2^ against ALL reflections. The weighted *R*-factor *wR* and goodness of fit *S* are based on *F*^2^, conventional *R*-factors *R* are based on *F*, with *F* set to zero for negative *F*^2^. The threshold expression of *F*^2^ > σ(*F*^2^) is used only for calculating *R*-factors(gt) *etc*. and is not relevant to the choice of reflections for refinement. *R*-factors based on *F*^2^ are statistically about twice as large as those based on *F*, and *R*- factors based on ALL data will be even larger.

### Fractional atomic coordinates and isotropic or equivalent isotropic displacement parameters (Å^2^)

**Table:**

	*x*	*y*	*z*	*U*_iso_*/*U*_eq_	
C1	0.32208 (15)	0.17035 (12)	0.77818 (9)	0.0137 (2)	
C2	0.42929 (16)	0.09481 (13)	0.85112 (10)	0.0168 (3)	
H2	0.534	0.119	0.8916	0.02*	
C3	0.38312 (16)	−0.01578 (13)	0.86458 (10)	0.0179 (3)	
H3	0.4572	−0.0668	0.9139	0.021*	
C4	0.22952 (17)	−0.05274 (12)	0.80657 (10)	0.0165 (3)	
C5	0.12331 (16)	0.02363 (13)	0.73350 (10)	0.0178 (3)	
H5	0.0186	−0.0005	0.693	0.021*	
C6	0.16804 (16)	0.13364 (13)	0.71908 (10)	0.0165 (3)	
H6	0.0942	0.1841	0.6691	0.02*	
C7	0.17416 (18)	−0.16732 (13)	0.81962 (11)	0.0222 (3)	
H7	0.0663	−0.1817	0.7798	0.027*	
C8	0.2598 (2)	−0.25211 (14)	0.88126 (13)	0.0269 (3)	
H8A	0.3683	−0.242	0.9226	0.032*	
H8B	0.2129	−0.3232	0.8842	0.032*	
C9	0.58097 (15)	0.32505 (12)	0.83389 (9)	0.0151 (2)	
C10	0.66759 (16)	0.24875 (14)	0.81638 (10)	0.0198 (3)	
H10	0.6167	0.1951	0.7644	0.024*	
C11	0.82745 (17)	0.25109 (14)	0.87468 (11)	0.0226 (3)	
H11	0.8857	0.1982	0.8631	0.027*	
C12	0.90255 (17)	0.33079 (15)	0.95012 (11)	0.0242 (3)	
H12	1.012	0.3324	0.99	0.029*	
C13	0.81748 (18)	0.40761 (15)	0.96690 (11)	0.0243 (3)	
H13	0.8689	0.4629	1.0178	0.029*	
C14	0.65719 (17)	0.40446 (13)	0.90988 (10)	0.0195 (3)	
H14	0.5996	0.4563	0.9227	0.023*	
C15	0.30934 (15)	0.32151 (12)	0.63898 (9)	0.0148 (2)	
C16	0.23401 (16)	0.42233 (13)	0.58296 (10)	0.0189 (3)	
H16	0.2133	0.4892	0.6084	0.023*	
C17	0.18938 (18)	0.42445 (15)	0.48962 (11)	0.0238 (3)	
H17	0.1383	0.4929	0.4515	0.029*	
C18	0.21944 (18)	0.32687 (15)	0.45237 (11)	0.0241 (3)	
H18	0.1895	0.3289	0.3889	0.029*	
C19	0.29308 (17)	0.22632 (14)	0.50744 (10)	0.0216 (3)	
H19	0.3136	0.1597	0.4817	0.026*	
C20	0.33703 (16)	0.22300 (13)	0.60052 (10)	0.0177 (3)	
H20	0.3859	0.1535	0.6379	0.021*	
P1	0.37156 (4)	0.31925 (3)	0.76149 (2)	0.01286 (8)	
Se1	0.270418 (17)	0.457326 (12)	0.793367 (11)	0.01969 (6)	

### Atomic displacement parameters (Å^2^)

**Table:**

	*U*^11^	*U*^22^	*U*^33^	*U*^12^	*U*^13^	*U*^23^
C1	0.0185 (6)	0.0122 (6)	0.0146 (6)	−0.0004 (5)	0.0119 (5)	−0.0007 (5)
C2	0.0169 (6)	0.0158 (6)	0.0193 (6)	0.0008 (5)	0.0113 (5)	0.0007 (5)
C3	0.0205 (6)	0.0144 (6)	0.0207 (7)	0.0033 (5)	0.0130 (6)	0.0033 (5)
C4	0.0233 (7)	0.0135 (6)	0.0183 (7)	−0.0012 (5)	0.0152 (6)	−0.0012 (5)
C5	0.0188 (6)	0.0190 (7)	0.0160 (6)	−0.0039 (5)	0.0102 (5)	−0.0018 (5)
C6	0.0195 (6)	0.0165 (6)	0.0135 (6)	−0.0002 (5)	0.0094 (5)	0.0010 (5)
C7	0.0278 (7)	0.0178 (7)	0.0266 (8)	−0.0043 (6)	0.0187 (6)	−0.0013 (6)
C8	0.0375 (9)	0.0177 (7)	0.0359 (9)	−0.0010 (6)	0.0271 (8)	0.0024 (6)
C9	0.0162 (6)	0.0147 (6)	0.0138 (6)	−0.0016 (5)	0.0082 (5)	0.0012 (5)
C10	0.0187 (6)	0.0215 (7)	0.0192 (7)	−0.0013 (5)	0.0108 (6)	−0.0024 (5)
C11	0.0188 (7)	0.0267 (8)	0.0235 (7)	0.0011 (6)	0.0127 (6)	0.0033 (6)
C12	0.0172 (6)	0.0288 (8)	0.0204 (7)	−0.0038 (6)	0.0070 (6)	0.0061 (6)
C13	0.0250 (7)	0.0234 (7)	0.0172 (7)	−0.0081 (6)	0.0074 (6)	−0.0018 (6)
C14	0.0236 (7)	0.0165 (6)	0.0170 (7)	−0.0024 (5)	0.0107 (6)	−0.0003 (5)
C15	0.0155 (6)	0.0165 (6)	0.0137 (6)	−0.0023 (5)	0.0091 (5)	0.0004 (5)
C16	0.0179 (6)	0.0190 (7)	0.0194 (7)	−0.0011 (5)	0.0103 (5)	0.0029 (5)
C17	0.0214 (7)	0.0273 (8)	0.0189 (7)	−0.0022 (6)	0.0093 (6)	0.0081 (6)
C18	0.0236 (7)	0.0352 (9)	0.0140 (7)	−0.0090 (6)	0.0110 (6)	0.0006 (6)
C19	0.0241 (7)	0.0269 (7)	0.0179 (7)	−0.0074 (6)	0.0145 (6)	−0.0060 (6)
C20	0.0197 (6)	0.0192 (6)	0.0157 (6)	−0.0024 (5)	0.0108 (5)	−0.0014 (5)
P1	0.01563 (15)	0.01136 (15)	0.01348 (16)	−0.00006 (11)	0.00938 (13)	−0.00031 (12)
Se1	0.02669 (9)	0.01456 (8)	0.02525 (9)	0.00329 (5)	0.01918 (7)	−0.00012 (5)

### Geometric parameters (Å, º)

**Table:**

C1—C2	1.3973 (19)	C11—C12	1.392 (2)
C1—C6	1.4005 (19)	C11—H11	0.95
C1—P1	1.8108 (14)	C12—C13	1.383 (2)
C2—C3	1.392 (2)	C12—H12	0.95
C2—H2	0.95	C13—C14	1.391 (2)
C3—C4	1.397 (2)	C13—H13	0.95
C3—H3	0.95	C14—H14	0.95
C4—C5	1.401 (2)	C15—C20	1.3959 (19)
C4—C7	1.4733 (19)	C15—C16	1.3981 (19)
C5—C6	1.3857 (19)	C15—P1	1.8198 (14)
C5—H5	0.95	C16—C17	1.396 (2)
C6—H6	0.95	C16—H16	0.95
C7—C8	1.322 (2)	C17—C18	1.388 (2)
C7—H7	0.95	C17—H17	0.95
C8—H8A	0.95	C18—C19	1.387 (2)
C8—H8B	0.95	C18—H18	0.95
C9—C14	1.396 (2)	C19—C20	1.394 (2)
C9—C10	1.399 (2)	C19—H19	0.95
C9—P1	1.8173 (14)	C20—H20	0.95
C10—C11	1.387 (2)	P1—Se1	2.1138 (4)
C10—H10	0.95		
			
C2—C1—C6	119.29 (12)	C13—C12—C11	119.86 (14)
C2—C1—P1	122.45 (10)	C13—C12—H12	120.1
C6—C1—P1	118.13 (10)	C11—C12—H12	120.1
C3—C2—C1	120.20 (13)	C12—C13—C14	120.49 (14)
C3—C2—H2	119.9	C12—C13—H13	119.8
C1—C2—H2	119.9	C14—C13—H13	119.8
C2—C3—C4	120.95 (13)	C13—C14—C9	120.00 (14)
C2—C3—H3	119.5	C13—C14—H14	120
C4—C3—H3	119.5	C9—C14—H14	120
C3—C4—C5	118.28 (13)	C20—C15—C16	119.64 (13)
C3—C4—C7	123.00 (13)	C20—C15—P1	120.28 (10)
C5—C4—C7	118.72 (13)	C16—C15—P1	120.07 (11)
C6—C5—C4	121.30 (13)	C17—C16—C15	119.78 (14)
C6—C5—H5	119.4	C17—C16—H16	120.1
C4—C5—H5	119.4	C15—C16—H16	120.1
C5—C6—C1	119.98 (13)	C18—C17—C16	120.19 (14)
C5—C6—H6	120	C18—C17—H17	119.9
C1—C6—H6	120	C16—C17—H17	119.9
C8—C7—C4	126.41 (15)	C19—C18—C17	120.23 (14)
C8—C7—H7	116.8	C19—C18—H18	119.9
C4—C7—H7	116.8	C17—C18—H18	119.9
C7—C8—H8A	120	C18—C19—C20	119.99 (14)
C7—C8—H8B	120	C18—C19—H19	120
H8A—C8—H8B	120	C20—C19—H19	120
C14—C9—C10	119.27 (13)	C19—C20—C15	120.14 (14)
C14—C9—P1	119.62 (11)	C19—C20—H20	119.9
C10—C9—P1	121.08 (11)	C15—C20—H20	119.9
C11—C10—C9	120.29 (14)	C1—P1—C9	105.68 (6)
C11—C10—H10	119.9	C1—P1—C15	104.47 (6)
C9—C10—H10	119.9	C9—P1—C15	107.07 (6)
C10—C11—C12	120.08 (14)	C1—P1—Se1	113.17 (4)
C10—C11—H11	120	C9—P1—Se1	112.96 (5)
C12—C11—H11	120	C15—P1—Se1	112.82 (5)
			
C6—C1—C2—C3	0.1 (2)	C16—C17—C18—C19	0.4 (2)
P1—C1—C2—C3	175.86 (11)	C17—C18—C19—C20	0.1 (2)
C1—C2—C3—C4	−0.7 (2)	C18—C19—C20—C15	−1.0 (2)
C2—C3—C4—C5	0.9 (2)	C16—C15—C20—C19	1.5 (2)
C2—C3—C4—C7	−178.22 (14)	P1—C15—C20—C19	−178.21 (11)
C3—C4—C5—C6	−0.6 (2)	C2—C1—P1—C9	14.26 (13)
C7—C4—C5—C6	178.61 (13)	C6—C1—P1—C9	−169.95 (11)
C4—C5—C6—C1	0.0 (2)	C2—C1—P1—C15	127.04 (12)
C2—C1—C6—C5	0.2 (2)	C6—C1—P1—C15	−57.17 (12)
P1—C1—C6—C5	−175.69 (11)	C2—C1—P1—Se1	−109.86 (11)
C3—C4—C7—C8	−4.9 (2)	C6—C1—P1—Se1	65.93 (11)
C5—C4—C7—C8	175.95 (16)	C14—C9—P1—C1	−115.72 (12)
C14—C9—C10—C11	0.8 (2)	C10—C9—P1—C1	62.47 (13)
P1—C9—C10—C11	−177.41 (11)	C14—C9—P1—C15	133.33 (11)
C9—C10—C11—C12	−1.0 (2)	C10—C9—P1—C15	−48.48 (13)
C10—C11—C12—C13	0.0 (2)	C14—C9—P1—Se1	8.54 (13)
C11—C12—C13—C14	1.1 (2)	C10—C9—P1—Se1	−173.27 (10)
C12—C13—C14—C9	−1.3 (2)	C20—C15—P1—C1	−42.60 (12)
C10—C9—C14—C13	0.4 (2)	C16—C15—P1—C1	137.66 (11)
P1—C9—C14—C13	178.59 (11)	C20—C15—P1—C9	69.18 (12)
C20—C15—C16—C17	−1.0 (2)	C16—C15—P1—C9	−110.55 (11)
P1—C15—C16—C17	178.70 (11)	C20—C15—P1—Se1	−165.94 (10)
C15—C16—C17—C18	0.1 (2)	C16—C15—P1—Se1	14.33 (12)

### Hydrogen-bond geometry (Å, º)

*Cg*1 and *Cg*2 are the centroids of the C1–C6 and C15–C20 rings, respectively.

**Table:**

*D*—H···*A*	*D*—H	H···*A*	*D*···*A*	*D*—H···*A*
C18—H18···*Cg*1^i^	0.95	2.62	3.383 (2)	137
C3—H3···*Cg*2^ii^	0.95	2.88	3.5889 (19)	133
C12—H12···*Cg*2^iii^	0.95	2.85	3.614 (2)	138

Symmetry codes: (i) *x*, −*y*−1/2, *z*−3/2; (ii) −*x*+1, *y*−1/2, −*z*+3/2; (iii) *x*+1, −*y*−1/2, *z*−1/2.
